# The clinicoradiological and pathological-molecular characteristics associated with spread through air spaces in stage IA invasive lung adenocarcinoma

**DOI:** 10.1186/s13244-026-02339-9

**Published:** 2026-06-24

**Authors:** Feng Xu, Xian Li, Wei-hua Zhao, Ji-wen Huo, Tian-you Luo, Qi Li

**Affiliations:** 1 Department of Radiology, The First Affiliated Hospital of Chongqing Medical University, Chongqing, China; 2 Department of Radiology, Guang an District People’s Hospital of Sichuan Province, Chengdu, China; 3 Department of Pathology, Chongqing Medical University, Chongqing, China

**Keywords:** Lung cancer, Computed tomography, Spreading through air spaces, Gene mutation

## Abstract

**Objectives:**

To investigate the clinicoradiological and pathological-molecular characteristics associated with spread through air spaces (STAS) in stage IA invasive lung adenocarcinoma (ILADC).

**Materials and methods:**

We retrospectively analyzed clinicoradiological data from 765 surgically resected stage IA ILADC patients. STAS-positive/-negative group comparisons identified independent predictors for model development, which were externally validated. Model-derived threshold stratified patients into risk groups for RFS analysis. Additionally, pathological and molecular tumor characteristics were compared between STAS-positive and STAS-negative cases.

**Results:**

Univariate analysis revealed significant differences in three clinical characteristics and eight radiological features between STAS-positive and STAS-negative cases (*p* < 0.05). Binary logistic regression identified CTR ≥ 90%, bronchial truncation, pleural retraction, and satellite nodules as independent predictors of STAS. The model yielded area under the curve (AUC) 0.823/accuracy 76.70% (training cohort) and AUC 0.829/accuracy 70.10% (external validation cohort). High-risk patients, as classified by the model, had significantly shorter RFS in the training and external validation cohorts (all *p* < 0.05). Additionally, STAS-positive tumors had a higher prevalence of micropapillary/solid-predominant growth and KRAS/ALK mutations, while EGFR mutations were more common in STAS-negative tumors (*p* < 0.05).

**Conclusion:**

The proposed model demonstrates potential in preoperatively identifying STAS and stratifying recurrence risk for patients with stage IA ILADC, which may assist in selecting appropriate candidates for sublobar resection. Furthermore, the distinct pathological and molecular characteristics of STAS-positive tumors offer valuable insights for guiding personalized adjuvant therapy.

**Critical relevance statement:**

This validated clinicoradiological model utilizes preoperative CT to predict STAS and recurrence risk, optimizing surgical decision-making. Furthermore, it explores these underlying pathological-molecular features associated with STAS, supporting personalized adjuvant therapy selection.

**Key Points:**

- Preoperative identification of STAS remains a clinical challenge in stage IA lung adenocarcinoma.
- Our clinicoradiological model can simultaneously predict STAS and stratify recurrence risk.
- STAS-positive cases can be characterized by micropapillary/solid patterns and KRAS/ALK mutations.
- These findings may assist with surgical decision-making and facilitate tailored adjuvant therapy selection.

**Graphical Abstract:**

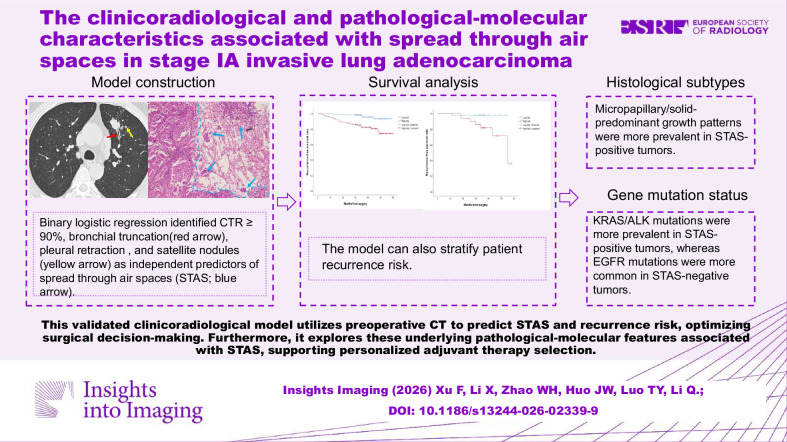

## Introduction

Lung adenocarcinoma (LADC), the most common histological subtype of lung cancer, continues to be a leading cause of cancer-related mortality globally [1, 2]. In 2015, the World Health Organization (WHO) classification of lung tumors formally designated spread through air spaces (STAS) as a distinct pattern of tumor invasion. STAS is defined by the presence of micropapillary clusters, solid nests, or single tumor cells within air spaces beyond the boundary of the primary tumor [3].

Surgical resection remains the cornerstone of treatment for early-stage LADC. For stage IA tumors, sublobar resection (SLR) is recommended based on several landmark randomized controlled trials, given its potential advantages over lobectomy. These advantages include superior preservation of lung function, minimized injury to lymph nodes and mediastinal structures, and lower perioperative morbidity [4–8]. However, STAS has emerged as a significant adverse prognostic factor, linked to an elevated risk of local recurrence following SLR [9–11]. This may be attributed to the dissemination of tumor cells within air spaces toward the surgical margin or ipsilateral lobe, potentially forming micrometastases and increasing recurrence risk [4, 9–11]. Therefore, accurate preoperative prediction of STAS is crucial for optimal surgical decision-making, underscoring the need for a reliable noninvasive predictive tool. Chest CT scans play a crucial role in the preoperative assessment of lung cancer. Although several clinical and CT features have been correlated with STAS in invasive lung adenocarcinoma (ILADC) [12, 13], the existing evidence is limited by small sample sizes. Moreover, the value of STAS prediction models based on clinicoradiological features in assessing recurrence risk among stage IA ILADC patients undergoing SLR remains insufficiently studied.

For elderly ILADC patients, or those with compromised cardiopulmonary function or high risk of perioperative complications, lobectomy may heighten mortality risk—rendering SLR the only feasible surgical alternative. Postoperative adjuvant therapy has been shown to improve outcomes in stage IA non-small cell lung cancer patients with high-risk pathological features for recurrence [14–16]. A deeper understanding of the pathological and molecular correlates of STAS could help identify STAS-positive patients who may benefit from adjuvant therapy after SLR. Nevertheless, the association between STAS and driver gene mutations is poorly understood.

Thus, this study aimed to develop and validate a predictive model for STAS in stage IA ILADC using clinical and imaging characteristics. We also evaluated the model’s performance in risk stratification for SLR candidates. Additionally, we investigated the pathological and molecular characteristics of STAS-positive tumors. We expect our findings to facilitate a more refined preoperative assessment of STAS and provide valuable evidence to guide clinical decision-making regarding the suitability of SLR.

## Materials and methods

### Patients

The institutional ethics committee (Approval Number: 2019-062) approved this study, and written informed consent was waived, given the retrospective design. A total of 886 patients with ILADC who received treatment from January 2019 to June 2022 were initially enrolled. The inclusion criteria were as follows: (1) clinical stage I ILADC (defined by the 9th Edition of the TNM Classification System) confirmed via comprehensive imaging (CT, brain MRI, whole-body PET/CT scans) and no metastatic evidence; (2) surgical pathological confirmation; (3) available preoperative chest CT images within 1 month prior to surgery. The interval between preoperative CT and subsequent lung resection ranged from 1 to 30 days, with a median of 11 (4, 12) days. Exclusion criteria included: (1) multiple primary lung cancers; (2) insufficient CT or pathological image quality for diagnosis; (3) antitumor therapy administered before CT scanning. After applying these criteria, 64 patients with multiple primary tumors, 19 with inadequate image quality, and 38 with prior antitumor therapy were excluded. The full eligible cohort of 765 patients (237 STAS-positive and 528 STAS-negative) was used to develop the STAS prediction model. An independent external validation cohort comprising 193 eligible patients (59 STAS-positive and 134 STAS-negative) from center 2, enrolled during the same period, was employed to externally validate the model.

**Fig. 1:**
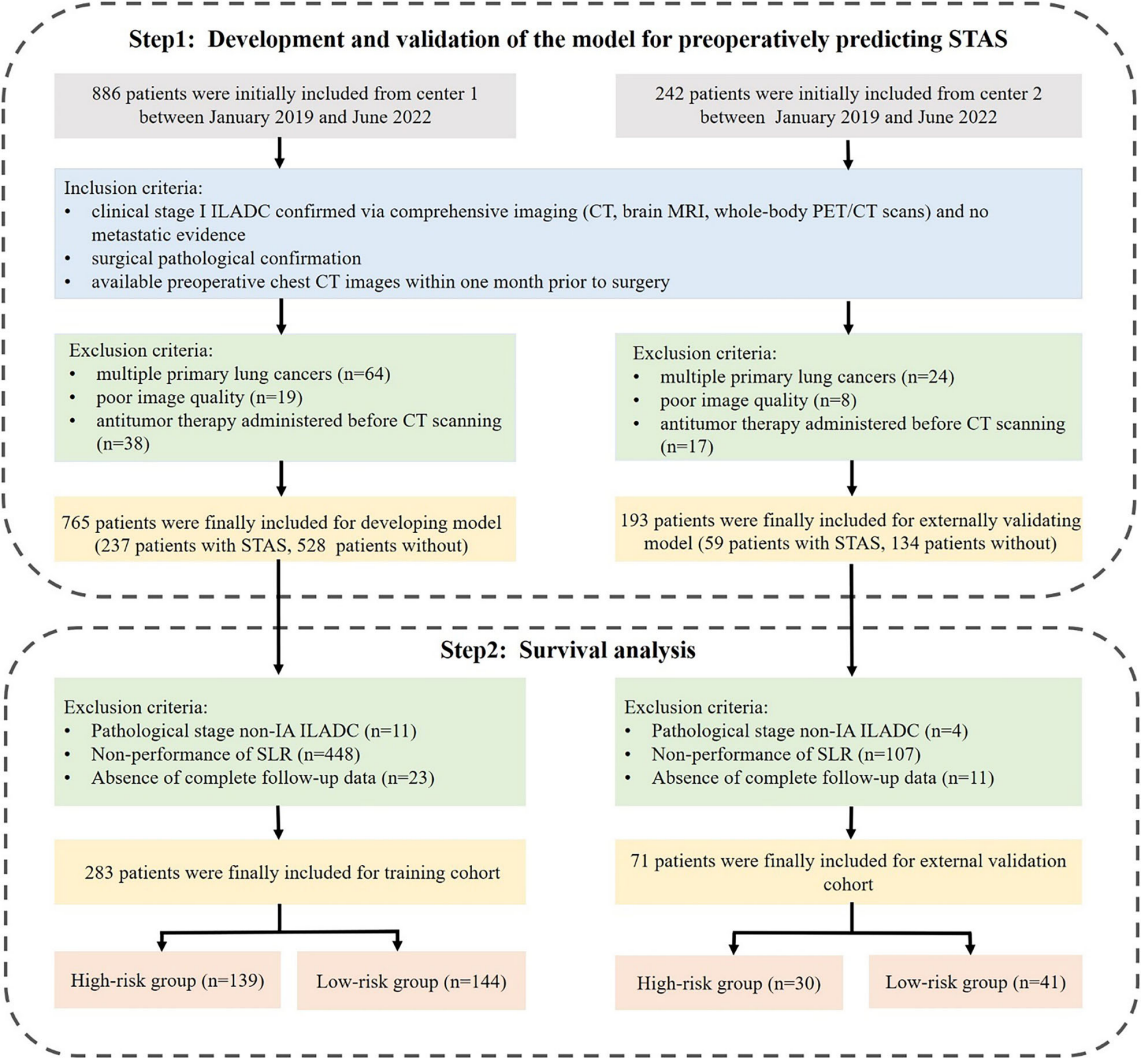
Flow diagram for patient selection in this study. ILADC, invasive lung adenocarcinoma; STAS, spread through air spaces; SLR, sublobar resection

Given that STAS strongly predicts local recurrence after SLR but not lobectomy, evaluating the model’s ability to stratify recurrence risk specifically within this subgroup is crucial to inform patient selection for SLR [9, 11]. Therefore, survival analysis was conducted in a cohort of 354 patients with pathological stage IA ILADC who underwent SLR and had complete follow-up data. Follow-up information was obtained during routine outpatient visits. In accordance with the institutional follow-up protocol, patients underwent chest CT at 3 and 6 months postoperatively, followed by surveillance CT scans every 6 months thereafter. These patients received regular postoperative CT surveillance for at least 3 years. Their surgical dates ranged from January 1, 2019, to June 10, 2022, and the final follow-up assessment was completed on October 10, 2025. Recurrence-free survival (RFS) was defined as the interval from the date of surgery to the date of tumor recurrence, death from any cause, or the last follow-up without evidence of disease progression. Recurrence was confirmed by imaging findings or, when available, histopathologic biopsy. This cohort comprised 283 patients from center 1 (training set) and 71 from center 2 (external validation set). The patient selection process is summarized in Fig. 1.

### CT scanning protocol

Preoperative chest CT scans were performed for all patients using one of the following scanners: Somatom Definition FLASH (Siemens Healthcare), Somatom Perspective (Siemens Healthcare), or Discovery CT750 HD (GE Healthcare). The scanning range extended from the first rib to the diaphragm. Key acquisition parameters included a tube voltage of 110–120 kVp, tube current of 50–250 mAs (with automatic tube current modulation), and a slice thickness/interval of 5 mm/5 mm. Among the cohort, 213 patients underwent contrast-enhanced CT imaging. Nonionic iodinated contrast agent (300 mg/mL iohexol (Omnipaque, GE Healthcare)) was injected via the antecubital vein using an automated injector at a dose of 1.5 mL/kg body weight and a flow rate of 3 mL/s, followed by a 45–50 mL saline flush. Arterial phase and delayed phase images were acquired at 30 s and 120 s after injection, respectively. All images were reconstructed with a thin-slice thickness/interval of 0.625–1.25 mm/0.625–1.25 mm and subsequently transferred to a Picture Archiving and Communication System workstation (Vue PACS, Carestream) for analysis.

### CT image interpretation

Two radiologists, with > 10 years of experience in thoracic radiology, were blinded to the related clinical data and independently evaluated CT images on a PACS workstation at each center. Discussion resolved any discrepancy in the assessment until a consensus was achieved. The lung and mediastinal windows were set as follows: window width, 1600 HU; window level, −600 HU; and window width, 400 HU; window level, 40 HU, respectively. The CT features of tumors were carefully evaluated as follows:

1. location (upper, middle, or lower lung lobe);
2. tumor size (maximum diameter of tumor measured from multiplanar recombination images on the lung window setting);
3. density (solid opacity or part-solid opacity);
4. consolidation tumor ratio (CTR) (the ratio of the maximum diameter of the solid component to that of the tumor);
5. margin (lobulation and spiculation);
6. internal characteristics (air space: air attenuation within the tumor, including cavity and pseudo-cavity; air bronchogram: air-filled bronchus within the tumor);
7. external characteristics (bronchial truncation: bronchial truncation at the edge of tumors; pleural retraction: linear structures connected between the tumor and pleura; peripheral fibrosis: caused by the tumor or preexisting fibrosis; halo sign: ill-defined ground-glass opacity around tumor; satellite nodules: nodules around tumor with the distance from tumor ≤ 3 cm).

For patients with sequential CT scans, image analyses were based on the latest CT data of tumors before surgery.

### Follow-up for survival analysis

The follow-up data for patients who underwent SLR were collected during outpatient visits. In accordance with our institution’s follow-up protocol, patients underwent chest CT scans at 3 and 6 months after surgery, with subsequent scans performed every 6 months thereafter. Recurrence-free survival (RFS) is defined as the duration from the date of surgery to either the date of tumor recurrence or death from any cause, or until the last follow-up without evidence of disease progression. The diagnosis of recurrence was confirmed through imaging examinations or, when feasible, biopsy. All patients received regular postoperative CT follow-up for a minimum of 3 years. Their surgical dates ranged from January 1, 2019, to June 10, 2022, and the final follow-up assessment was completed on October 10, 2025.

### Histochemical examination and gene mutation testing

All surgically resected specimens were fixed in 10% formalin, placed onto paraffin blocks, sectioned into 5-mm-thick sections, and stained with hematoxylin and eosin. Two experienced pathologists, blinded to the patients’ clinical data, collaboratively evaluated the specimens and reached their conclusions through consensus. STAS was considered to exist when the micropapillary clusters, solid nests, or single cells spread within the air spaces beyond the edge of the main tumor according to the 2015 WHO classification [3]. The percentage of each growth pattern (lepidic, acinar, papillary, micropapillary, solid, etc.) within the tumor was assessed semi-quantitatively at 5% increments, and the pattern with the highest proportion was defined as the predominant pattern. For genomic mutation analysis, tumor DNA was extracted from surgical resection specimens. Genetic alterations, including mutations or fusions of EGFR, ALK, KRAS, BRAF, HER2, ROS1, MET, PIK3CA, RET, and NRAS, were detected using commercially available kits based on the amplification refractory mutation system–real-time polymerase chain reaction (ARMS‑qPCR) or next-generation sequencing (NGS) platforms from Amoy Diagnostics.

### Statistical analysis

Statistical analyses were performed using Statistical Package for the Social Sciences version 26.0 (IBM Corporation). The intraclass correlation coefficient (ICC) was used to evaluate the diagnostic consistency of CT characteristics between the two observers, and an ICC value of > 0.75 indicated good agreement. For normally distributed continuous variables presented as mean ± standard deviation, comparisons between the two groups were conducted using the independent samples Student’s *t*-test. For non-normally distributed continuous variables expressed as median (25th and 75th percentiles), the Mann–Whitney U test was applied. For categorical variables reported as counts and percentages, the chi-square test was utilized for group comparisons. Binary logistic regression analysis was performed to identify independent predictors of STAS using clinical and CT characteristics that differed significantly between the two groups. Prior to model construction, multicollinearity among candidate predictors was assessed using the variance inflation factor (VIF). Variables with a VIF > 5 were considered to indicate significant multicollinearity and were subsequently excluded from the regression analysis. The regression model was built using the enter method, and its performance was evaluated using the area under the curve (AUC), accuracy, sensitivity, and specificity. A *p*-value of < 0.05 was considered to indicate statistical significance. Based on the regression model, receiver operating characteristic (ROC) analysis was performed using the predicted probabilities. The optimal cutoff probability for stratifying patients into high- and low-risk groups was determined using the Youden index. Survival curves were constructed using the Kaplan–Meier method and compared using the log-rank test.

## Results

### Development and validation of the preoperative STAS prediction model

#### Comparison of clinical features between the two groups

Table 1 summarizes the baseline clinical characteristics of the included patients. Among the total cohort of 765 patients, 237 (31.0%) had STAS-positive tumors (131 males and 106 females; median age 64 [54, 69] years [range: 27–83]), while 528 (69.0%) had STAS-negative tumors (193 males and 335 females; median age 62 [55, 69] years [range: 30–86]). Male sex, a history of smoking, and elevation of CEA were significantly more common in the STAS-positive group (all *p* < 0.05). However, no significant differences were observed between the two groups in terms of age and other tumor marker levels (all *p* > 0.05).

**Table 1:** Comparison of clinical features between the two groups

Characteristics	STAS-positive group (*n* = 237)	STAS-negative group (*n* = 528)	*p*-value
Gender			< 0.001^a^
Male	131 (55.27%)	193 (36.55%)	
Female	106 (44.73%)	335 (63.45%)	
Age (years)			0.494^b^
Median	64 (54, 69)	62 (55, 69)	
Range	27–78	30–86	
Smoking history			< 0.001^a^
Smokers	98 (41.35%)	130 (24.62%)	
Non-smokers	139 (58.65%)	398 (75.38%)	
Tumor marker levels			
Elevation of CEA	33 (13.92%)	47 (8.90%)	0.036^a^
Elevation of CK19	53 (22.36%)	124 (23.48%)	0.734^a^
Elevation of neuron-specific enolase	10 (4.22%)	16 (3.03%)	0.401^a^
Elevation of SCCAg	11 (4.64%)	11 (2.08%)	0.050^a^
Elevation of pro-grp	23 (9.70%)	50 (9.47%)	0.919^a^

*STAS* spread through air spaces, *CEA* carcinoembryonic antigen, *CK19* cytokeratin 19 fragment, *SCCAg* squamous cell carcinoma antigen, *pro-grp* pro-gastrin-releasing peptide

^a^ Chi-squared test

^b^ Mann–Whitney U test

#### Comparison of CT features between the two groups

Interobserver agreement for all CT features was excellent. The ICC values between the two observers were as follows: location, 1.000; tumor size, 0.927; lobulation, 0.947; spiculation, 0.904; density, 0.955; consolidation-to-tumor ratio (CTR), 0.936; air space, 0.922; air bronchogram, 0.935; bronchial amputation, 0.924; pleural retraction, 0.958; peripheral fibrosis, 0.931; halo sign, 0.929; and satellite nodules, 0.946. All ICC values were statistically significant (all *p* < 0.001).

**Fig. 2:**
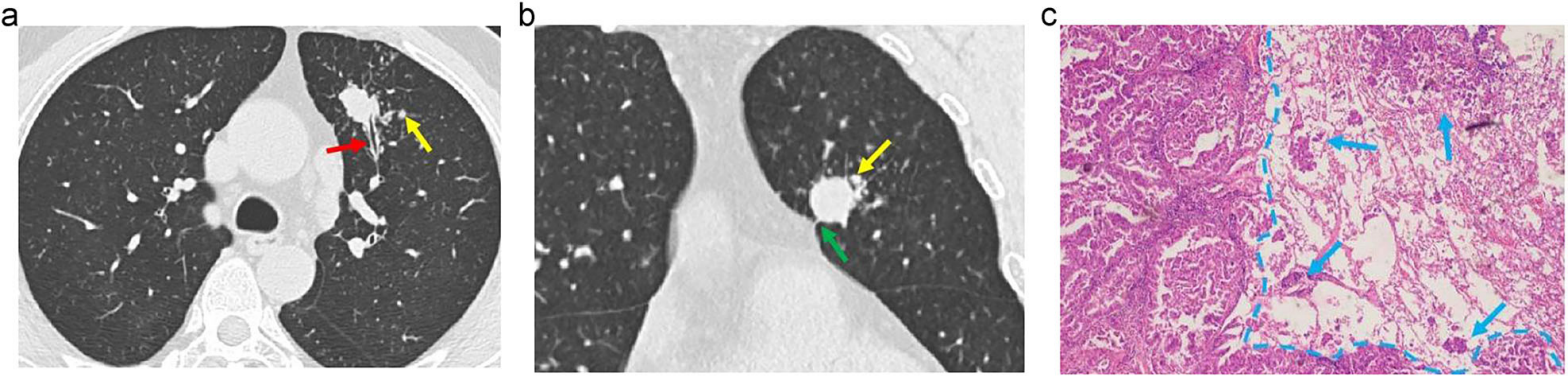
ILADC in a 61-year-old man. **a**, **b** Transverse and coronal CT images of the lung window setting reveal a solid nodule with lobulation, bronchial truncation (red arrow), pleural retraction (green arrow), and satellite nodules (yellow arrow) in the left upper lobe. **c** Photomicrography (hematoxylin and eosin staining; magnification × 40) reveals ILADC primarily exhibiting a micropapillary growth pattern, with STAS in the peritumoral area (blue arrows)

**Fig. 3:**
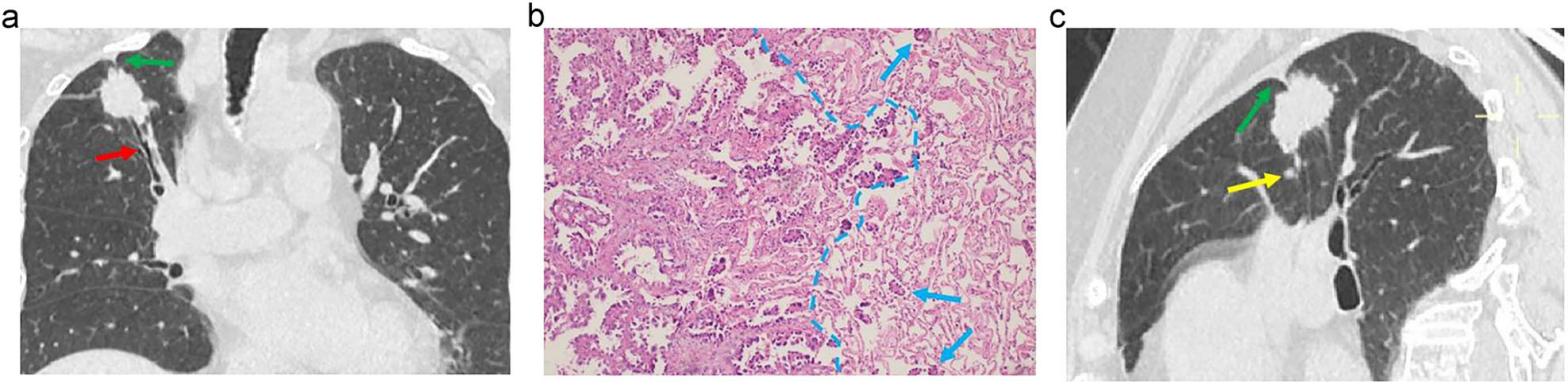
ILADC in an 80-year-old woman. **a**, **b** Transverse and sagittal CT images of the lung window setting reveal a solid nodule with a lobulation, bronchial truncation (red arrow), pleural retraction (green arrow), and satellite nodules (yellow arrow) in the right upper lobe. **c** Photomicrography (hematoxylin and eosin staining; magnification × 100) reveals ILADC primarily exhibiting a micropapillary growth pattern, with STAS in the peritumoral area (blue arrows)

**Fig. 4:**
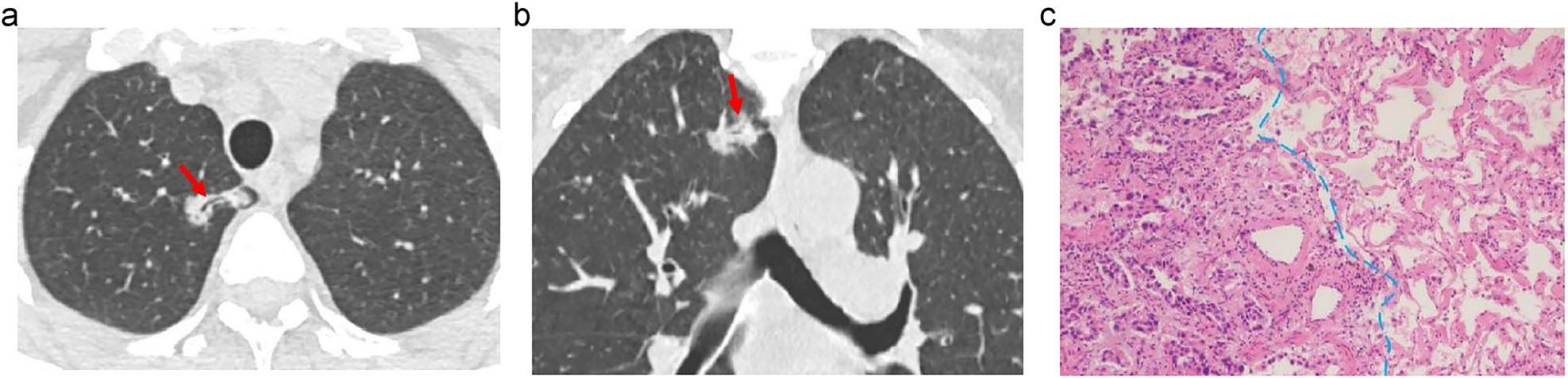
LADC in a 51-year-old woman. **a**, **b** Transverse and coronal CT images of the lung window setting reveal a part-solid nodule (CTR < 90%) with an air bronchogram (red arrow) in the right upper lobe. **c** Photomicrography (hematoxylin and eosin staining; magnification × 100) reveals ILADC with an acinar growth pattern. The tumor border is marked by a blue dashed line, and no STAS is observed in the peritumoral area

The median tumor size was 20 (14, 24) mm (range: 6–30 mm) in the STAS-positive group and 18 (14, 20) mm (range: 6–30 mm) in the STAS-negative group. The CTR was significantly higher in STAS-positive tumors (median: 100% [95%, 100%]; range: 18%–100%) compared to STAS-negative tumors (median: 71% [42%, 100%]; range: 8%–100%; *p* < 0.001). The optimal CTR cutoff for predicting STAS was 90%, yielding an AUC of 0.758, with a sensitivity of 82.3% and specificity of 63.4%. As summarized in Table 2, solid opacity, CTR ≥ 90%, lobulation, spiculation, bronchial truncation, pleural retraction, peripheral fibrosis, and satellite nodules were significantly more common in STAS-positive tumors, whereas air bronchogram was more frequent in STAS-negative tumors (all *p* < 0.05). However, no significant differences were observed between the two groups in terms of tumor location, tumor size, air space, and halo sign (all *p* > 0.05) (Figs. 2–4).

**Table 2:** Comparison of CT features between the two groups

Characteristics	STAS-positive group (*n* = 237)	STAS-negative group (*n* = 528)	*p*-value
Location			0.116^a^
Upper lobe	127 (53.59%)	315 (59.66%)	
Middle/lower lobe	110 (46.41%)	213 (40.34%)	
Tumor size (mm)			0.116^b^
Median	20 (14, 24)	18 (14, 20)	
Range	6–30	6–30	
Density			< 0.001^a^
Solid opacity	176 (74.26%)	170 (32.20%)	
Part-solid opacity	61 (25.74%)	358 (67.80%)	
CTR			< 0.001^a^
≥ 90%	195 (82.28%)	193 (36.55%)	
< 90%	42 (17.72%)	335 (63.45%)	
Lobulation	207 (87.34%)	353 (66.86%)	< 0.001^a^
Spiculation	132 (55.70%)	146 (27.65%)	< 0.001^a^
Air space	77 (32.49%)	170 (32.20%)	0.936^a^
Air bronchogram	96 (40.51%)	304 (57.58%)	< 0.001^a^
Bronchial truncation	79 (33.33%)	29 (5.49%)	< 0.001^a^
Pleural retraction	173 (73.00%)	252 (47.73%)	< 0.001^a^
Peritumoral fibrosis	152 (64.14%)	266 (50.38%)	< 0.001^a^
Halo sign	8 (3.38%)	9 (1.70%)	0.147^a^
Satellite nodules	35 (14.76)	9 (1.70%)	< 0.001^a^

*STAS* spread through air spaces, *CTR* consolidation tumor ratio

^a^ Chi-squared test

^b^ Mann–Whitney U test

#### Development and validation of the logistic regression model

Among the nine clinical and CT features that differed significantly between the two groups, multicollinearity diagnostics revealed notable collinearity between tumor density and CTR ≥ 90% (VIF > 5). After excluding tumor density, the remaining predictors showed no significant multicollinearity, with VIF values as follows: CTR ≥ 90% (1.435), lobulation (1.243), spiculation (1.320), air bronchogram (1.116), bronchial truncation (1.301), pleural retraction (1.293), peritumoral fibrosis (1.363), and satellite nodules (1.074). Binary logistic regression analysis demonstrated that CTR ≥ 90%, bronchial truncation, pleural retraction, and satellite nodules were identified as independent predictors of STAS in patients with stage IA ILADC (Table 3). The probability of STAS was calculated using the following logistic function: P = 1 / (1 + e^(2.686 - 1.485 × CTR ≥ 90% - 1.143 × bronchial truncation - 0.917 × pleural retraction - 1.803 × satellite nodules)^). This model achieved an AUC of 0.823, with an accuracy of 76.7%, sensitivity of 87.8%, and specificity of 64.0%. In the external validation cohort, the model yielded an AUC of 0.829, accuracy of 71.0%, sensitivity of 84.8%, and specificity of 64.9%. The baseline characteristics of the external validation cohort are provided in Supplementary Table 1.

**Table 3:** Binary logistic regression analysis

Variables	B	Standard error	wald	Odds ratio (95% CI)	*p*-value
CTR ≥ 90%	1.485	0.227	42.770	4.416 (2.829–6.892)	< 0.001
Bronchial truncation	1.143	0.271	17.786	3.136 (1.844–5.334)	< 0.001
Pleural retraction	0.917	0.221	17.277	2.503 (1.624–3.858)	< 0.001
Satellite nodules	1.803	0.448	16.194	6.065 (2.521–14.591)	< 0.001
Constant	−2.686	0.289	86.552	0.068	< 0.001

*CTR* consolidation tumor ratio, *CI* confidence interval

### Survival analysis of the model

**Fig. 5:**
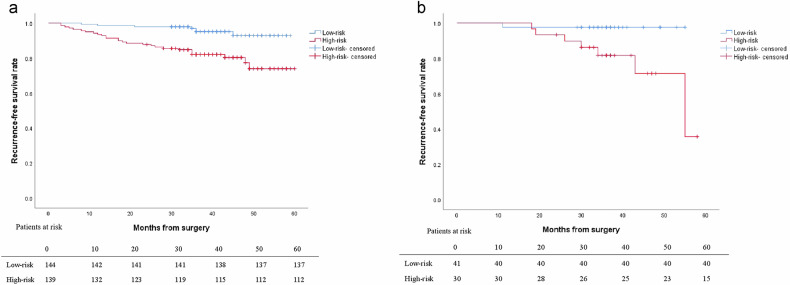
The Kaplan–Meier curves depicting recurrence-free survival for high- and low-risk patients in the training cohort (**a**) and the external validation cohort (**b**)

Follow-up data were collected from both centers until October 10, 2025. A total of 354 patients with pathological stage IA ILADC who underwent SLR and had complete follow-up data were included in the survival analysis. The median age was 62 (54, 69) years (range: 30–83). The postoperative follow-up duration ranged from 11 to 65 months, with a median of 40 (35, 49) months. Using the optimal probability threshold (*p* = 0.207) derived from the training cohort model, patients were classified into high-risk (*p* > 0.207) and low-risk (*p* ≤ 0.207) groups. In the training cohort (*n* = 283 from center 1), 139 patients were categorized as high-risk and 144 as low-risk. The RFS rates were 80.57% in the high-risk group and 95.14% in the low-risk group. Kaplan–Meier analysis showed significantly shorter RFS in the high-risk group (*p* < 0.05), with a hazard ratio (HR) of 4.319 (95% CI: 1.881–9.920; *p* = 0.001). In the external validation cohort (*n* = 71 from center 2), 30 patients were high-risk and 41 were low-risk. The RFS rates were 76.67% and 97.56%, respectively. Consistent with the training cohort, the high-risk group had significantly worse RFS (*p* < 0.05), with an HR of 8.694 (95% CI: 1.057–71.512; *p* = 0.044) (Fig. 5).

### Pathological and molecular characteristics associated with STAS

Among all 680 patients, the micropapillary/solid‑predominant growth pattern was significantly more common in STAS‑positive tumors, while the lepidic/acinar/papillary‑predominant subtypes were more frequent in STAS‑negative tumors (all *p* < 0.05) (Table 4 and Figs. 2–4). Among the 484 patients with available genetic mutation data, *EGFR* mutation was more prevalent in STAS‑negative tumors, whereas *KRAS* mutation and *ALK* fusion were significantly enriched in STAS‑positive tumors (all *p* < 0.05) (Table 4). However, no significant differences were observed between the two groups in mutations or fusions involving *BRAF*, *HER2*, *ROS1*, *MET*, *PIK3CA*, *RET*, and *NRAS* (all *p* > 0.05).

**Table 4:** Comparison of pathological-molecular features between the two groups

Histological subtypes (*n* = 680)	STAS-positive group (*n* = 237)	STAS-negative group (*n* = 528)	*p*-value
Micropapillary/solid-predominant subtypes	218 (91.98%)	124 (23.48%)	< 0.001^a^
Lepidic/acinar/papillary-predominant subtypes	19 (8.02%)	404 (76.52%)	

Gene mutation status (*n* = 484)	STAS-positive group (*n* = 135)	STAS-negative group (*n* = 349)	*p*-value
EGFR			< 0.001^a^
Present	68 (50.37%)	261 (74.79%)	
Absent	67 (49.63%)	88 (25.21%)	
KRAS			0.037^a^
Present	14 (10.37%)	17 (4.87%)	
Absent	121 (89.63%)	332 (95.13%)	
ALK			< 0.001^a^
Present	15 (11.11%)	6 (1.72%)	
Absent	120 (88.89%)	343 (98.28%)	
BRAF			0.124^a^
Present	5 (3.70%)	4 (1.15%)	
Absent	130 (96.30%)	345 (98.85%)	
HER2			0.531^a^
Present	2 (1.48%)	9 (2.58%)	
Absent	133 (98.52%)	340 (97.42%)	
ROS1			0.355^a^
Present	3 (2.22%)	3 (0.86%)	
Absent	132 (97.78%)	346 (99.14%)	
MET			0.337^a^
Present	0 (0%)	4 (1.15%)	
Absent	135 (100%)	345 (98.85%)	
PIK3CA			1.000^a^
Present	1 (0.74%)	2 (0.57%)	
Absent	134 (99.26%)	347 (99.43%)	
RET			1.000^a^
Present	1 (0.74%)	2 (0.57%)	
Absent	134 (99.26%)	347 (99.43%)	
NRAS			1.000^a^
Present	0 (0%)	0 (0.00%)	
Absent	135 (100%)	349 (100%)	

*STAS* spread through air spaces

^a^ Chi-squared test

## Discussion

STAS has recently been recognized as a distinct pattern of tumor invasion in LADC and is considered a major risk factor for recurrence in early-stage ILADC patients treated with SLR [9–11]. Consequently, accurate preoperative identification of STAS is crucial for developing optimal treatment strategies. This study comprehensively investigated the clinicoradiological, pathological, and molecular characteristics associated with STAS in stage IA ILADC, yielding several key findings.

First, we developed an effective predictive model integrating clinical and preoperative CT characteristics to identify STAS in ILADC patients meeting SLR criteria. The model identified several independent predictors of STAS, including CTR ≥ 90%, bronchial truncation, pleural retraction, and satellite nodules, with an AUC of 0.823. In recent years, the CTR has emerged as a key imaging biomarker and independent prognostic factor in early-stage lung cancer [17, 18]. In our study, CTR was the most predictive CT-derived feature, with an optimal cutoff value of 90%, yielding a sensitivity of 82.3% and specificity of 63.4%, consistent with Kim et al’s report [12]. Given its straightforward measurement on CT, CTR represents a valuable imaging biomarker for guiding therapeutic decisions. Our results indicate that bronchial truncation should raise suspicion for STAS. Pathologically, this feature reflects tumor invasion and destruction of airway structures. The compromised airways may serve as conduits for tumor cell dissemination, facilitating spread to adjacent alveolar spaces or distal bronchioles, ultimately leading to STAS [19]. Pleural retraction, caused by fibrotic retraction or tumor invasion, was a significant predictor of STAS in our study, a finding consistent with the retrospective analysis of 83 patients by Toyokawa et al [20]. Additionally, air bronchogram, which reflects preserved bronchi within the tumor, was found to be inversely associated with STAS [21]. Satellite nodules were confirmed as a valuable CT feature associated with STAS. In our cohort, they were present in 14.76% (35/237) of STAS-positive tumors, compared to only 1.70% (9/528) of STAS-negative cases. This finding aligns with Zhang et al [22], who reported a comparable incidence (7.43%, 9/121) of satellite nodules in STAS-positive tumors. In contrast, Qi et al [23] observed no significant association between satellite nodules and STAS, despite noting a higher frequency in STAS-positive LADC—a discrepancy that may reflect variations in patient selection or sample size across studies. We propose that satellite nodules may represent a macroscopic radiological correlate of STAS. External validation of the prediction model demonstrated moderate performance and generalizability, yielding an AUC of 0.829. However, its specificity for preoperative STAS prediction in stage IA ILADC remained modest (64.0% and 64.9% in the development and external validation cohorts, respectively), indicating a non-negligible false-positive rate. In clinical practice, such errors could lead to unnecessary lobectomy over SLR, resulting in avoidable loss of lung parenchyma. While current guidelines permit lobectomy for clinical stage IA disease and recommend SLR in appropriately selected patients, improving model specificity is essential to minimize overtreatment risk. Achieving this goal will require integrating multi-parametric data with deeper biological insights into STAS.

Moreover, this study showed that patients stratified as high-risk by the model had significantly shorter RFS and may benefit more from lobectomy than from SLR. In summary, the proposed model facilitates noninvasive characterization of tumor aggressiveness, estimation of recurrence risk, and support of personalized surgical decision-making for patients with clinical stage IA ILADC. We envision that this model could be implemented in clinical practice as an AI-powered clinical decision-support tool integrated into existing diagnostic workflows. Using this system, radiologists or clinicians can input CT features, including consolidation-to-tumor ratio, bronchial truncation, pleural retraction, and satellite nodules, to generate an individualized STAS probability. This quantitative output may assist surgeons in determining the optimal extent of resection, particularly in clinically equivocal cases.

Furthermore, we investigated the pathological-molecular features of STAS-positive ILADC. This study showed that STAS-positive tumors were strongly associated with the micropapillary and solid-predominant subtypes, consistent with prior reports [24, 25]. This finding further strengthens the well-established correlation between STAS positivity and poor prognosis. Moreover, our analysis revealed that EGFR mutations were more frequent in STAS-negative tumors, while KRAS mutations and ALK fusions were more prevalent in STAS-positive tumors, aligning with earlier studies [26–28]. Elucidating these STAS-associated pathological-molecular profiles may offer valuable insights to guide the development of personalized postoperative adjuvant therapies. While NCCN guidelines do not routinely recommend adjuvant therapy for stage IA NSCLC, emerging evidence suggests survival benefits for patients with actionable driver alterations (e.g., EGFR, KRAS, ALK) [14–16]. Our findings demonstrated a higher prevalence of KRAS mutations and ALK fusions in STAS-positive tumors, suggesting a link between STAS and specific oncogenic drivers. This association carries important clinical implications, especially for patients with limited cardiopulmonary reserve or advanced age, for whom lobectomy poses increased risk and SLR represents the only feasible surgical option. In such contexts, STAS positivity coupled with KRAS/ALK alterations may define a subgroup that could benefit from adjuvant therapy, even at stage IA.

This study has several limitations. First, its retrospective design may introduce selection bias, despite efforts to include consecutive eligible patients. Second, CT images were interpreted by radiologists from two institutions; despite the use of predefined criteria and blinded assessments, inter-observer variability and differences in institution-specific imaging protocols may have affected the reproducibility of the imaging features. Third, no comparative analysis was performed regarding the clinical, imaging, and pathological-molecular characteristics between solid and subsolid ILADC with STAS, which warrants further investigation. Finally, the interpretation of CT features remains inherently subjective. Future studies incorporating automated feature extraction via machine learning may improve diagnostic consistency and further refine and extend the findings of the present study.

In conclusion, our predictive model demonstrates a good capacity for the preoperative identification of both STAS and high-risk patients for recurrence after SLR in clinical stage IA ILADC, thereby potentially informing surgical decision-making regarding the selection of SLR. Moreover, the distinct pathological-molecular signature of STAS-positive tumors paves the way for personalizing postoperative adjuvant treatment strategies.

## Supplementary information

ELECTRONIC SUPPLEMENTARY MATERIAL

## Data Availability

All data generated or analyzed during the study are included in the published paper.

## Abbreviations

AUC: Area under the curve
CI: Confidence interval
CT: Computed tomography
CTR: Consolidation tumor ratio
ICC: Intraclass correlation coefficient
ILADC: Invasive lung adenocarcinoma
LADC: Lung adenocarcinoma
RFS: Recurrence-free survival
SLR: Sublobar resection
STAS: Spreading through air spaces
VIF: Variance inflation factor
