# Supplementary material for: The clinicoradiological and pathological-molecular characteristics associated with spread through air spaces in stage IA invasive lung adenocarcinoma
Source: Insights Imaging. 2026 Jun 24;17:171. doi: 10.1186/s13244-026-02339-9 (PMC13294424; doi:10.1186/s13244-026-02339-9)
Supplement: Supplementary file 1 — ELECTRONIC SUPPLEMENTARY MATERIAL [file 13244_2026_2339_MOESM1_ESM.pdf]

The Clinicoradiological and Pathological-molecular Characteristics Associated with Spread Through Air Spaces in Stage IA Invasive Lung Adenocarcinoma

ELECTRONIC SUPPLEMENTARY MATERIAL

Supplementary Table S1 . The baseline characteristics of the external validation cohort

**Clinical features**	**STAS-positive group** (n=59)	**STAS-negative group** (n=134)	***p*-value**
Sex			0.173 [a]
Male	30 (50.85%)	54 (38.28%)	
Female	29 (49.15%)	80 (61.72%)	
Age (years)			0.107 [b]
Average	23.10 ± 18.20	23.10 ± 18.20	
Range	41-83	31-81	
Smoking history			0.208 [a]
Smokers	24 (40.68%)	42 (31.34%)	
Non-smokers	35 (59.32%)	92 (68.66%)	
Tumor markers			
Elevation of CEA	10 (16.95%)	8 (5.97%)	0.016 [a]
Elevation of CK19	8 (13.56%)	28 (20.90%)	0.228 [a]
Elevation of Neuron-specific Enolase	0 (0.00%)	3 (2.24%)	0.247 [a]
Elevation of SCCAg	0 (0.00%)	5 (3.73%)	0.133 [a]

Elevation of Pro-grp	6 (10.17%)	5 (3.73%)	0.0768 [a]
Location			0.214 [a]
upper lobe	30 (50.85%)	81 (60.45%)	
middle/ lower lobe	29 (49.15%)	53 (39.55%)	
Tumor size (mm)			0.142 [c]
Median	19 (15, 24)	18 (14, 20)	
Range	8–30	7–30	
Density			< 0.001[a]
Solid opacity	43 (72.88%)	41 (60.45%)	
Part-solid opacity	16 (27.12%)	93 (39.55%)	
CTR			< 0.001 [a]
≥ 90%	45 (76.27%)	45 (33.58%)	
< 90%	14 (23.73%)	89 (66.42%)	
Lobulation	52 (88.14%)	77 (57.46%)	
Spiculation	33 (55.93%)	41 (30.60%)	
Air space	20 (33.90%)	48 (35.82%)	
Air bronchogram	26 (44.07%)	80 (59.70%)	
Bronchial amputation	21 (35.59%)	6 (4.48%)	
Pleural retraction	47 (79.66%)	75 (55.97%)	
Peritumoral fibrosis	42 (71.19%)	72 (53.73%)	
Halo sign	3 (5.08%)	3 (2.24%)	
Satellite nodules	10 (16.95%)	3 (2.24%)	

Abbreviations: STAS, spread through air spaces; CEA, carcinoembryonic antigen;CK 19 ,cytokeratin 19 fragment; SCCAg,Squamous cell carcinoma antigen; pro-grp,pro-gastrin-releasing peptide.

[a] Chi-squared test

[b] Two-independent-samples Student's *t*-test

[c] Mann-Whitney U test
